# Phenolic Secondary Metabolites in *Aldrovanda vesiculosa* L. (Droseraceae)

**DOI:** 10.3390/molecules30183746

**Published:** 2025-09-15

**Authors:** Magdalena Wójciak, Ireneusz Sowa, Maciej Strzemski, Marzena Parzymies, Magdalena Pogorzelec, Piotr Stolarczyk, Bartosz J. Płachno

**Affiliations:** 1 Department of Analytical Chemistry, Medical University of Lublin, 4a Chodzki St., 20-093 Lublin, Poland; 2 Institute of Horticultural Production, University of Life Sciences in Lublin, 20-950 Lublin, Poland; 3 Department of Hydrobiology and Protection of Ecosystems, University of Life Sciences in Lublin, 20-950 Lublin, Poland; 4 Department of Botany, Physiology and Plant Protection, Faculty of Biotechnology and Horticulture, University of Agriculture in Kraków, 29 Listopada 54 Ave., 31-425 Krakow, Poland; 5 Department of Plant Cytology and Embryology, Institute of Botany, Faculty of Biology, Jagiellonian University, 9 Gronostajowa St., 30-387 Krakow, Poland

**Keywords:** aquatic plants, carnivorous plants, ellagic acid, flavonoids, gallic acid, hydroplumbagin glucoside, liquid chromatography, phytochemistry, plumbagin, waterwheel plant

## Abstract

*Background: Aldrovanda vesiculosa* L. is a small aquatic plant that produces snap traps for capturing zooplankton prey. *Aldrovanda* belongs to the family Droseraceae, which is well known for the production of secondary metabolites (especially naphthoquinones). However, compared to other species in this family (*Drosera* and *Dionaea*), *A. vesiculosa* has been very poorly studied in terms of metabolites. *The Aim*: To fill this gap in knowledge, we investigated what secondary metabolites are present in the shoots of these plants. A hypothesis was tested stating that there are more metabolites in the younger (apical) parts of the shoots, which protect them from herbivores. *Methods*: Shoots of *A. vesiculosa* were collected, and the plant material was extracted with methanol, followed by 80% methanol or pure acetone using the accelerated solvent extraction method. The phytochemical profile was established using UPLC-DAD-(ESI)-MS. *Results*: *A. vesiculosa* shoots contained gallic acid and its derivatives, ellagic acid and its derivatives, flavonoids, and naphthoquinones (plumbagin and hydroplumbagin hexoside). A gradient (apical–basal) of gallic acid, ellagic acid, plumbagin, and hydroplumbagin hexoside was observed in the shoots. Meanwhile, the total flavonoid content did not differ between the middle and apical parts but was significantly lower in the basal part. In general, the lowest concentrations of metabolites were found in the basal part and the highest in the apical part, with the exception of total flavonoids. The number of free flavonoid aglycones was significantly higher in the middle part, whereas the apical part was dominated by glycoside derivatives.

## 1. Introduction

The genus *Aldrovanda* L., together with the genera *Drosera* L. and *Dionaea* Sol. ex J.Ellis, belongs to the family Droseraceae Salisb., order Nepenthales Bercht. & J.Presl. All these plants are carnivorous plants; however, *Aldrovanda* and *Dionaea* form snap traps, unlike *Drosera*, which produces adhesive traps for prey catching. The *Aldrovanda* genus contains a few fossil species and only one recent *Aldrovanda vesiculosa* L. species (waterwheel plant), which is widely distributed as an azonal–aquatic plant in the Old World [1,2]. *Aldrovanda* is considered a tertiary (Paleogene) element, and the only one recent *A. vesiculosa* is a relict species. *A. vesiculosa* is also extremely rare and threatened with extinction [3,4,5]. Research on *A. vesiculosa* mainly focuses on the adaptation of this species to carnivory, e.g., [6,7,8,9,10]. The most prominent prey organisms of *A. vesiculosa* are crustaceans (Cladocera, Ostracoda, and Copepoda), but Mollusca, mites, and insects are also trapped [11]. The mechanics of how the traps work are extremely interesting and have been deeply studied, especially since *A. vesiculosa* traps are among the fastest-moving plant organs [12,13,14,15,16]. The structure of the traps and glands of *A. vesiculosa* has also been studied [17,18,19].

Recently, Wójciak et al. [20] reviewed contemporary research on metabolites in Nepenthaceae and Droseraceae and showed that these plants may be a source of phenolic acids and their derivatives (gallic, protocatechuic, chlorogenic, ferulic, *p*-coumaric, gallic, hydroxybenzoic, vanillic, syringic, and caffeic acids, as well as vanillin); flavonoids (myricetin, quercetin, and kaempferol derivatives), including anthocyanins (delphinidin-3-O-glucoside, cyanidin-3-O-glucoside, cyanidin); naphthoquinones (e.g., plumbagin, droserone, and 5-O-methyl droserone), and volatile organic compounds. Schlauer et al. [21] showed that the acetogenic naphthoquinones plumbagin and ramentaceone are valuable phytochemical markers that aid in the delimitation, identification, and classification of Droseraceae taxa, especially in the *Drosera* genus. Also, chemometric data have been used to show the relationships between 16 *A. vesiculosa* populations from various sites, including four continents [22]. Secondary metabolites from Droseraceae have great biological potential in terms of antibacterial, antifungal, antioxidant, anti-inflammatory, and anticancer activities. Among these metabolites, naphthoquinones are of particular interest, especially plumbagin (5-hydroxy-2-methyl-1,4-naphthoquinone), which is the best-studied naphthoquinone found in Droseraceae. It exhibits pronounced antimicrobial activity against Gram-positive and Gram-negative bacteria, as well as several fungal pathogens. In addition, plumbagin shows significant cytotoxic potential against various cancer cell lines. However, despite its promising pharmacological profile, plumbagin has also been reported to be mutagenic and genotoxic [23,24].

A review by Wójciak et al. [20] shows that there is still scarce information on the chemistry of some carnivorous plants; in particular, the genus *Aldrovanda* is poorly explored. Plumbagin was found in *A. vesiculosa* by Zenk et al. [25], Culham and Gornall [26], and Adamec et al. [27]. Recently, shoots and turions of *A. vesiculosa* were studied regarding the occurrence of fatty acids [28].

To fill the gap in knowledge, we investigated which secondary metabolites are present in the shoots of these plants. A hypothesis was tested that there are more metabolites in the younger (apical) parts of the shoots, which protect them from herbivores. We identified secondary metabolites in in vitro-cultured plants because *A. vesiculosa* requires specific growing conditions [29], so it was easier to control the growth conditions in vitro than to grow plants in outdoor containers. In addition, in vitro plant cultivation serves as preparation for future production in bioreactors.

## 2. Results

### 2.1. Phytochemical Characterization Based on UPLC-DAD-(ESI)-MS Analysis

**Figure 1:**
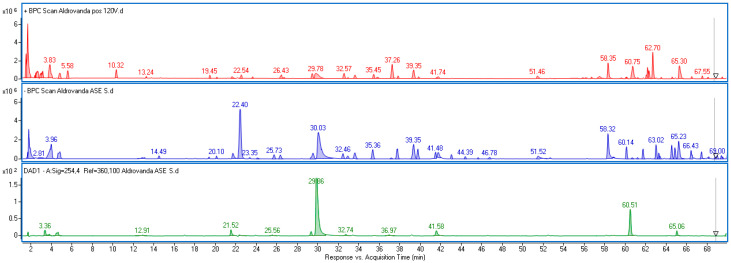
Base peak chromatograms (BPCs) of *Aldrovanda vesiculosa* extract obtained in positive ionization (red line) and negative ionization (blue line), along with DAD chromatogram recorded at λ = 254 nm (green line).

Polyphenolic acids and flavonoids were identified in negative ionization mode based on MS data and fragmentation patterns obtained using different ionization energies. Furthermore, positive ionization mode was used to verify the results. Spectral data were compared with appropriate standards when available. Otherwise, the compounds were tentatively identified based on the literature concerning Droseraceae [30]. Empirical formulas were established using the MassHunter software (ver. 10.0), with an acceptable difference between estimated and theoretical formulas not exceeding 5 ppm. UV–Vis spectra in a range of 200–600 nm were also recorded, and compounds were assigned to particular classes of metabolites based on their characteristic absorption maxima as follows: gallic acid derivatives showed a maximum at 270–275 nm; ellagic acid exhibited a characteristic spectrum with two absorption maxima at 252–255 nm (sharp) and 362–365 nm; flavonoids displayed absorption maxima at 250–260 nm and 350–365 nm. Plumbagin, the main naphthoquinone in *A. vesiculosa*, exhibited two absorption maxima at 267 nm and 418 nm. Examples of the MS and UV–Vis spectra of the main identified components are shown in Figure S1 (Supplementary Materials). The base peak chromatogram (BPC) obtained for *Aldrovanda* extract, both in positive and negative ionization modes, along with the DAD chromatogram registered at 254 nm, is shown in Figure 1.

Gallic acid derivatives showed a common ion at *m*/*z* = 169.014, corresponding to free gallic acid (GA), and *m*/*z* = 125 as a result of decarboxylation of GA. Among them, different isomeric forms of galloylhexoside (*m*/*z* = 331) were the most abundant. Ellagic acid (EA) and its derivatives showed a common ion at *m*/*z* = 300.999. The presence of free ellagic acid and methylellagic acid was confirmed by comparison with the relevant standards, while the other derivatives of EA were tentatively identified based on MS and UV–Vis spectra. From the flavonoid class, kaempferol 3-O-glucoside and subsequently, kaempferol were the predominant components. They were identified based on the common ion at *m*/*z* [M − H]^−^ = 285, typical for aglycone, and the radical aglycone ion at *m*/*z* [M − H]^−^ = 284 [31]. In turn, quercetin and quercetin 3-O-glucoside, which were present in significantly lower amounts, showed a common ion at *m*/*z* [M − H]^−^ = 301 (aglycone) and a radical ion at *m*/*z* [M − H]^−^ = 300 [31]. Myricetin (*m*/*z* [M − H]^−^ = 317) and its glucoside (*m*/*z* [M − H]^−^ = 463) were also identified; however, their concentrations were low. The retention times, MS data, and UV–Vis spectra of the aforementioned flavonoids matched those of the corresponding standards. An unknown flavonoid, exhibiting a UV–Vis absorption maximum characteristic for kaempferol and showing *m*/*z* [M − H]^−^ = 609.1463 and *m*/*z* [M + H]^+^ = 611.1607 (formula: C_27_H_30_O_16_), displayed fragment ions in positive mode at *m*/*z* [M + H]^+^ = 449.10845 (C_21_H_22_O_11_) and 287.0547 (C_15_H_12_O_6_) (Figure S2). Based on these data, it was tentatively identified as kaempferol dihexoside.

Naphthoquinones were primarily represented by plumbagin, which was detected with positive ionization (*m*/*z* = 189.05). Furthermore, the derivative of plumbagin—hydroplumbagin hexoside and hydroplumbagin dihexoside with *m*/*z* = 351.108 and 513.160 [M − H]^−^, respectively, and a fragment ion at *m*/*z* = 189 corresponding to free hydroplumbagin were identified. The presence of unstable glycosidic forms of naphthoquinones has previously been documented in *Drosera* species [32].

Mass data for the phenolic components found in *A. vesiculosa*, ordered according to retention times, are summarized in Table 1.

### 2.2. Comparison of the Metabolite Content in Different Parts of the Plant

Two different extraction solvents were used to assess the metabolite content in *A. vesiculosa* shoots in order to maximize the yield of specific groups of metabolites. Methanol (followed by 80% methanol) was used for the extraction of flavonoids and phenolic acids, as highly polar solvents are recommended for polyphenols, whereas acetone was used for the isolation of naphthoquinones following the method of Dwivedi et al. [37]. Such a selection of solvents, combined with the use of accelerated solvent extraction, allows for exhaustive extraction of metabolites from individual groups (Figure S3).

**Figure 2:**
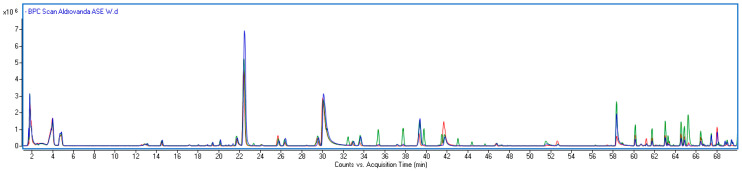
Overlapped BPC chromatograms of *Aldrovanda vesiculosa* samples from different plant regions (red line—base part, green line—middle part, blue line—apical part).

As can be seen in Figure 2, the qualitative profiles of extracts from different parts of the plant were similar; however, differences in peak intensity were observed for some components, suggesting a different quantitative profile. Table 2 presents the results of the quantification of the main identified constituents in the base, middle, and apical parts of the *Aldrovanda* shoot.

Quantitative analysis of the main phenolic metabolites in different parts of *Aldrovanda vesiculosa* shoots revealed clear differences in compound concentrations depending on the shoot segment. The total content of gallic acid derivatives increased from the basal (B) to the apical part (A) of the shoot, with values of 5.67, 6.38, and 7.07 mg/g dry weight (DW), respectively. Free gallic acid ranged from 1.81 mg/g in B to 2.73 mg/g in A, while galloyhexose content was relatively stable (3.13–3.36 mg/g). Digalloylhexose reached the highest level in the middle and apical sections.

The total content of ellagic acid derivatives was highest in the apical part (21.29 mg/g). Free ellagic acid was the predominant compound in this group, with concentrations of ca 14 mg/g in the B and M sections and 19.3 mg/g in the apical part. Methylellagic acid showed a decreasing gradient from the base (2.38 mg/g) to the apex (0.70 mg/g). Glucosides of ellagic and methylellagic acid, as well as dimethylellagic acid hexoside, were present in smaller amounts and showed no clear directional trend.

The total flavonoid content, expressed in µg/g DW, was markedly higher in the middle and apical parts (1609.34 and 1568.6 µg/g, respectively) than in the basal part (471.04 µg/g). Kaempferol 3-O-glucoside was the dominant flavonoid, showing a pronounced increase from B (340.8 µg/g) to M (968.2 µg/g) and A (1070.4 µg/g). Free kaempferol peaked in the middle section (516.6 µg/g). Interestingly, the middle part tended to accumulate free aglycones, whereas in the apical section, glycoside derivatives were more abundant. Naphthoquinones were represented by plumbagin and dihydroplumbagin and its glycosides. Plumbagin concentration increased progressively from 6.7 mg/g in the basal section to 8.7 mg/g in the apical section. Hydroplumbagin glucoside followed a similar trend, reaching its maximum in the apical part. Its concentration immediately after extract preparation was even higher than that of plumbagin; however, this component was labile and underwent degradation during storage of the extract at room temperature (Figure S4).

## 3. Discussion

An important feature of *A. vesiculosa*’s life cycle and growth is its continuous apical growth during the growing season, with progressive aging and decomposition at the base [38]. This species is very efficient in nutrient reutilization from senescent shoots. Nutrients from the dying part of the shoot are delivered to the apical part, which is characterized by rapid growth [7]. The linear shoot of *A. vesiculosa* also shows polarity in physiological terms. Adamec [39] demonstrated a change in chlorophyll content and a progressive decrease in photosynthetic rate in tissues (leaves and traps) located further from the apical part. According to Šimura et al. [40], zeatin cytokinins predominate in the apical parts of *A. vesiculosa* shoots, with their concentrations decreasing basipetally. Our results indicate that there is also polarity in terms of phytochemicals in the shoots of *A. vesiculosa*. We observed a gradient (apical–basal) of gallic acid, ellagic acid, kaempferol dihexoside, plumbagin, and dihydroplumbagin hexoside in *A. vesiculosa* shoots. In general, the lowest concentrations of metabolites were found in the basal part and the highest in the apical part, with the exception of total flavonoids, for which the difference between the middle section and apical part was not statistically significant. Interestingly, the number of free flavonoid aglycones was significantly higher in the middle part, whereas the apical part was dominated by glycoside derivatives.

It is worth noting that, to date, only a few studies have focused on the phytochemical composition of *A. vesiculosa*, and the available data exclusively concern the naphthoquinone group. The presence of plumbagin (without quantitative data) in *A. vesiculosa* was mentioned by Culham et al. [26]. Adamec, in turn, compared plumbagin content in *Aldrovanda vesiculosa* plants of different origins and found that its levels vary significantly depending on growth conditions, ranging from none to 0.7% in the basal part and from 1.2% to 2.4% in the apical part (HPLC data) [27]. In our investigation, the amount of plumbagin was within the lower range (0.67–0.87%), which is likely due to differences in cultivation conditions. Interestingly, the major compound identified in *Aldrovanda* species was another member of this group, specifically dihydroplumbagin hexoside. The presence of dihydroderivatives of naphthoquinones was previously detected in *Drosera* species [32,41].

The composition of the cultivation medium, particularly the type and concentration of plant growth regulators, macronutrients, micronutrients, and other additives, may strongly influence the phytochemical profile and secondary metabolite production in in vitro-cultured plants, for example, adjusting the nutrient balance, nitrogen forms and ratios, and the levels of auxins and cytokinins can significantly alter the synthesis of various phenolic compounds, flavonoids, and other bioactive compounds. These were shown using various species, e.g., *Withania somnifera* (L.) Dunal [42], *Hypericum perforatum* L. [43,44], *Schisandra chinensis* (Turcz.) Baill. [45], and *Lychnis flos-cuculi* L. [46]. Also, light conditions may have an impact on metabolite production [47], e.g., *Ruta graveolens* L. [48], *Moluccella laevis* L. [49], *Salvia yangii* (BT Drew) [50], and *Lychnis flos-cuculi* L. [46]. However, Adamec et al. [27] showed that plumbagin content was similar in both sun-adapted and shade-adapted *Aldrovanda* plants. More information is available for *Dionaea* and *Drosera* cultivation. Siatkowska et al. [51] cultivated *Drosera binata* Labill. and *D. peltata* Thunb. on two media differing in mineral composition, sucrose content, and pH. These authors observed differences in secondary metabolism between plants of the same species grown on different media. Boonsnongcheep et al. [52] showed the effect of artificial LED lights on the plumbagin level of *Drosera burmannii* Vahl and *D. indica* L. According to Putalun et al. [53], methyl jasmonate, yeast extract, and chitosan stimulated plumbagin production in *D. burmanii*. The elicitation-based method also increased the production of phenolic compounds in *Dionaea muscipula* [54,55].

In the case of plumbagin content, our results are consistent with the observations of Adamec et al. [27], who also found accumulation of plumbagin in apical parts of shoots. The compounds are probably synthesized intensively in the apical part of the shoot, where intensive growth occurs and new traps are formed. However, it cannot be ruled out that some compounds are withdrawn from older parts and transported to the youngest ones. This would be beneficial in terms of the plant’s resource conservation. However, according to Adamec et al. [27], plumbagin is released from aging tissues into the surrounding medium (unfortunately, there are no results or methodology for these studies). The concentration of actively metabolic compounds is likely to protect the meristem and the youngest traps, which would otherwise be vulnerable to being eaten by animals. Tokunaga et al. [56,57], who found that *Dionaea muscipula* Ellis accumulates a large amount of plumbagin, proposed that the Droseraceae family possesses a universal defensive mechanism against predators based on the synthesis and accumulation of naphthoquinones. Dávila-Lara et al. [58] showed that carnivorous *Nepenthes* plants use naphthoquinones against herbivores. In the case of *A. vesiculosa*, a similar mechanism was also suggested by Adamec et al. [27]. However, it should be noted that *Aldrovanda* turions are consumed by birds due to high starch content [38]. It is worth noting that Adamec et al. [27] found plumbagin in the *Aldrovanda* turions. So, the presence of plumbagin does not prevent birds from eating turions. It remains an open question whether the presence of plumbagin can limit consumption, for example, by restricting the number of turions a bird can safely ingest. Despite advances in chemical analysis and the structure of turions [17,59,60,61], there is still a lack of comprehensive information on all secondary metabolites in turions. Therefore, turions should also be examined in detail in the future.

Plumbagin exhibits significant antimicrobial activity against various bacteria and fungi [62,63]. Adamec et al. [27] suggested that plumbagin may provide *Aldrovanda* with resistance to microorganisms. Despite the accumulation of plumbagin, *Aldrovanda* plants are attacked by several *Phytopythium* and *Pythium* (Oomycetes) species [64]. It is interesting to see to what extent these pathogens have developed resistance to plumbagin.

It is worth emphasizing that our work is the first report providing both qualitative and quantitative data on secondary metabolites from the polyphenol group in *A. vesiculosa* species. Notably, in addition to plumbagin, this plant material is also a rich source of kaempferol, kaempferol-3-O-glucoside, and ellagic acid.

The limitations of the study are as follows: Our investigation focused on polar and semi-polar metabolites from the group of phenolic compounds. However, as shown by the chromatogram recorded over a wide time window (Figure S5), *Aldrovanda vesiculosa* also contains numerous lipophilic compounds that warrant further investigation. Also, it is known that in vitro-grown plants often exhibit a much simpler metabolite profile compared to plants collected from natural habitats; thus, further research on *Aldrovanda* should also include wild plants from natural conditions.

## 4. Materials and Methods

### 4.1. Plant Material and Sample Collection

The plant materials were individuals of *Aldrovanda vesiculosa* collected from 4 peat bog lakes situated in eastern Poland. They were Lake Długie (N51°27′17.18″; E23°10′12.60″) and Lake Łukie (N51°24′40.30″; E23°04′56.73″), in Poleski National Park, and Lake Orchowe (N51°29′27.63″; E23°34′26.12″) and Lake Płotycze (N51°23′38.42″; E23°36′59.84″) in Sobiborski Landscape Park. The shoots were transported to the tissue culture laboratory in plastic containers containing lake water.

In the laboratory, the shoots were defoliated and cut into 2 cm pieces, followed by disinfection. As a first step, they were rinsed under running tap water and then shaken in the water with a drop of detergent (Ludwik, GRUPA INCO S.A., Warsaw, Poland) twice for 10 min.; then, the shoots were immersed in 70% ethanol (Chempur, Piekary Śląskie, Poland) for 10 s. At the next step, surface sterilization was performed by shaking the shoots in sodium hypochlorite water solution (NaOCl, Chempur, Poland) at a concentration of 0.25% for five minutes. The disinfected shoots were rinsed 3 times in sterilized distilled water and placed individually in tubes containing 10 mL of the liquid medium, which consisted Murashige and Skoog (MS) [65] macro- and microelements supplemented with 0.1 mg·dm^−3^ thiamine (vit. B_1_), 0.5 mg·dm^−3^ pyridoxine (vit. B_6_), 0.5 mg·dm^−3^ niacine (vit. PP), 2.0 mg·dm^−3^ glycine, 100 mg·dm^−3^ myo-inositol, and 20 g·dm^−3^ sucrose. All the components were diluted 10 times. The medium composition was selected on the basis of the previous research [66]. Medium pH was established at 5.5. The medium was steam-sterilized for 21 min. at a temperature of 121 °C under 1 hPa of pressure. After the initiation of tissue culture, the plants were cultivated in 450 mL jars containing 200 mL of the medium and covered with semi-transparent plastic lids at 5-week intervals. Each time, only the green parts were used. The flasks with explants were placed in a growing room at a temperature of 25 °C, with a 16 h photoperiod. The source of light was fluorescent, using daylight lamps with 30 µmol·m^−2^·s^−1^ light intensity (Philips TLC 58W/84; Philips, Polska).

**Figure 3:**
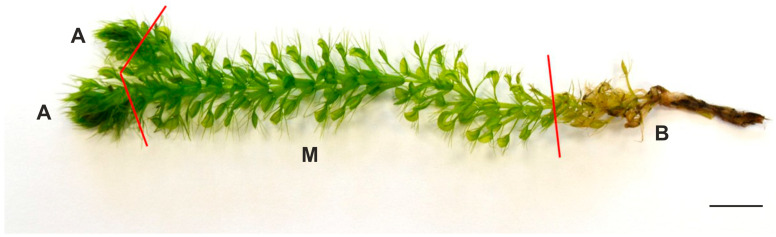
Plant material used in the present study. Shoot of *Aldrovanda vesiculosa*: apical part (A), middle part (M), basal part (B); bar = 1 cm.

To conduct the analysis, shoots (113 plants) were taken out of the media, washed under distilled water, and fragmented into apical parts (fragments with apex), middle parts (green with leaves with mature developed traps), and basal parts (yellow-brown fragments, which are typically discarded from plants) (Figure 3). The obtained fragments were dried off on a paper towel, oven-dried at a temperature of 30 °C for 48 h, and then pooled together and powdered.

### 4.2. Extraction of Plant Metabolites

Accelerated Solvent Extraction (ASE): Approximately 200 mg of powdered plant material was extracted using a Dionex ASE 350 extractor (Thermo Fisher Scientific Inc., Sunnyvale, CA, USA) with methanol, followed by 80% methanol (for polyphenolic compounds) or twice pure acetone (for plumbagin). Extraction conditions were set at 65 °C, with a static time of 10 min and a dynamic extraction time of 5 min. The resulting extracts were combined, centrifuged, and adjusted to a final volume of 50 mL. The mixture was then filtered through a 0.2 µm syringe filter. Two independent extraction procedures were performed for each extrahent.

### 4.3. Analysis

Chromatographic Conditions: An ultra-high performance liquid chromatograph (UHPLC) Infinity Series II with a DAD detector, an Agilent 6224 ESI/TOF mass detector (Agilent Technologies, Santa Clara, CA, USA), and an RP18 reversed-phase column (10 cm × 2.1 mm i.d., 1.9 µm particle size) were used to achieve the separation. Water with 0.05% formic acid (solvent A) and acetonitrile with 0.05% formic acid (solvent B) at a flow rate of 0.2 mL/min were the mobile phases. The gradient program was as follows: 0–8 min from 98% A to 93% A (from 2% to 7% B), 8–15 min from 93% A to 88% A (from 7% to 12% B), 15–29 min from 88% A to 85% A (from 12% to 15% B), 29–40 min from 85% A to 80% A (from 15% B to 20% B), 40–50 min from 80% A to 75% A (from 20% B to 25% B), and 50–60 min from 75% A to 55% A (from 25% B to 45% B). The thermostat temperature was 30 °C. DAD chromatograms were recorded from 200 to 600 nm. MS-ESI Parameters: Drying gas temperature, 325 °C; drying gas flow, 8 L min^−1^; nebulizer pressure, 30 psi; capillary voltage, 3500 V; skimmer voltage, 65 V; and slicer voltage, 200 V and 260 V for negative mode and 120 V for positive mode. Ions were acquired from 100 to 1200 *m*/*z*. MS and UV–Vis spectrum main components identified in *A. vesiculosa* extracts are provided in Figures S1 and S2. The injection volume for both samples and standards was 2 µL. The number of replicates was three for each solution. Quantification was carried out using calibration curves prepared for standard solutions. The concentration range and calibration equations are provided in the Supplementary Material (Table S1). MS-grade acetonitrile and analytical standards, including gallic acid, epigallocatechin gallate, myricetin 3-O-glucoside, ellagic acid, quercetin 3-O-glucoside, kaempferol 3-O-glucoside, quercetin, kaempferol, and plumbagin, were purchased from Sigma-Aldrich (St. Louis, MO, USA).

## 5. Conclusions

Our study is the first report providing qualitative and quantitative data on secondary metabolites in *Aldrovanda vesiculosa*. In addition to plumbagin, this plant is also rich in gallic acid, ellagic acid, and their derivatives, as well as in flavonoids, including kaempferol and its glycosides, quercetin, and quercetin glucoside. Furthermore, it has been shown that there was polarity in the occurrence of secondary metabolites in the shoots of *A. vesiculosa*. We observed a gradient (apical–basal) of gallic acid, ellagic acid, kaempferol dihexoside, plumbagin, and hydroplumbagin glucoside in *A. vesiculosa* shoots. In general, the lowest concentrations of metabolites were found in the basal part and the highest in the apical part, with the exception of total flavonoids. The content of free flavonoid aglycones was significantly higher in the middle part, whereas the apical part was dominated by glycoside derivatives.

## Figures and Tables

**Table 1:** Mass data extracted from main peaks found in *Aldrovanda vesiculosa* extract.

Rt (min)	[M − H]^−^/(Fragments)	Error (ppm)	[M + H]^+^	Error (ppm)	Formula	UV–Vis (nm)	Identified	Ref
1.90	331.06793 (169,125)	2.59	333.08196	1.01	C_13_H_16_O_10_	218,280	Galloyl hexoside	[33]
3.96	331.06730 (169,125)	0.69	333.08208	1.38	C_13_H_16_O_10_	218,280	Galloyl hexoside	[33]
4.79	169.01417 (125)	−0.45	171.02889	0.53	C_7_H_6_O_5_	215,270	Gallic acid	str, [34]
8.54	331.06709 (169,125)	0.06	-		C_13_H_16_O_10_	218,280	Galloyl hexoside	[33]
12.91	483.07725 (331,169)	−1.61	-		C_20_H_20_O_14_	218,280	Digalloyl hexoside	[33]
13.06	397.1136 (173)	−1.06	399.12908	1.27	C_18_H_22_O_10_	220,275	Unknown	
14.49	513.16042 (351)	−1.84	515.17603	0.22	C_23_H_30_O_13_	230,305	Dihydroplumbagin dihex.	[32]
17.16	625.10608 (463,299)	2.30	-		C_26_H_26_O_18_	255,370	Ellagic acid dihexoside	[34]
18.04	625.10535 (463,299)	1.74	-		C_26_H_26_O_18_	-	Ellagic acid dihexoside	[34]
19.39	353.12433 (173)	0.39	355.13916	1.17	C_17_H_22_O_8_	-	Unknown	
20.10	609.14637	0.43	611.16078	0.19	C_27_H_30_O_16_	265,365	Kaempferol dihexoside	[35]
21.32	457.07711 (169,125)	−1,15	-		C_22_H_18_O_11_	-	Epigallocatechin gallate	str, [34]
21.70	463.05150 (301)	−0.68	465.06688	1.11	C_20_H_16_O_13_	255,360	Ellagic acid glucoside	[33]
22.40	351.10802 (189)	−1.48	353.12420	3.14	C_17_H_20_O_8_	230,305	Dihydroplumbagin hex.	[32]
23.35	561.18252 (515)	0.05	-		C_24_H_34_O_15_	-	Unknown	
24.17	477.06643 (315)	−2.16	-		C_21_H_18_O_13_	-	Methylellagic acid hex.	[33,34]
25.73	365.08786 (203,175)	0.15	-		C_17_H_18_O_9_	250,280,380	Unknown	
26.42	479.08243 (316)	−1.43	-		C_21_H_20_O_13_	255,370	Myricetin 3-O-glucoside	str, [33,36]
29.50	477.06834 (315)	1.83	479.08346	3.02	C_21_H_18_O_13_	250,370	methylellagic acid hex.	[33,34]
30.03	301.00033	4.43	303.01365	0.35	C_14_H_6_O_8_	255,370	Ellagic acid	str, [33]
32.46	635.34872	−1.32	-		C_27_H_56_O_16_	-	Unknown	
33.01	491.08192 (301)	−2.43	493.09789	0.45	C_22_H_20_O_13_	250,375	Dimethylellagic acid hex.	[33]
33.61	463.08231 (301)	−4.58	465.10287	0.25	C_21_H_20_O_12_	255,365	Quercetin 3-O-glucoside	str, [36]
35.36	679.37499	−1.15	-		C_29_H_60_O_17_	-	Unknown	
37.78	723.40212	0.18	-		C_31_H_64_O_18_	-	Unknown	
39.35	447.09514 (284)	4.14	449.10854	1.57	C_21_H_20_H_11_	265,365	Kaempferol 3-O-glucoside	str, [36]
39.80	767.42832	0.15	-		C_33_H_68_O_19_	-	Unknown	
41.24	317.03023	−0.19	-		C_15_H_10_O_8_	255,370	Myricetin	str, [36]
41.76	315.01482	0.57	317.02984	2.05	C_15_H_8_O_8_	250,375	Methylellagic acid	[33]
51.52	301.03451	−2.87	303.05043	1.66	C_15_H_10_O_7_	255,370	Quercetin	str, [33,36]
58.32	285.04111	2.27	287.05589	3.06	C_15_H_10_O_6_	265,365	Kaempferol	str, [33,36]
60.71	-	-	189.0549 [M + H]^+^	1.49	C_11_H_8_O_3_	270,420	Plumbagin	str, [32]

hex—hexoside; “-”—spectrum unclear or lack of spectrum; str—identification was confirmed using standard; the other compounds were tentatively identified based on mass data, fragmentation pattern, UV–Vis spectra, and comparison with the literature.

**Table 2:** The results of quantification of the phenolic metabolites found in *Aldrovanda* extract from the base (B), middle (M), and apical parts (A) of the shoots expressed per gram of dried material (±SD).

Components	B	M	A
Gallic acid and derivatives (mg/g)			
Gallic acid	1.81 ± 0.16 ^c^	2.17 ± 0.24 ^b^	2.73 ± 0.31 ^a^
Galloyl hexosides (total) ^1^	3.28 ± 0.35 ^a^	3.13 ± 0.25 ^a^	3.36 ± 0.38 ^a^
Digalloyl hexosides ^1^	0.58 ± 0.03 ^b^	1.08 ± 0.06 ^a^	0.98 ± 0.01 ^a^
Total:	5.67 ± 0.26 ^c^	6.38 ± 0.29 ^b^	7.07 ± 0.31 ^a^
ellagic acid and derivatives (mg/g)			
Ellagic acid glucoside ^2^	0.52 ± 0.02 ^c^	0.72 ± 0.04 ^a^	0.60 ± 0.03 ^b^
Methylellagic acid glucoside ^2^	0.41 ± 0.02 ^b^	0.54 ± 0.03 ^a^	0.42 ± 0.04 ^b^
Ellagic acid	14.25 ± 1.4 ^b^	14.51 ± 1.56 ^b^	19.31 ± 2.17 ^a^
Dimethylellagic acid hexoside ^2^	0.22 ± 0.02 ^b^	0.25 ± 0.03 ^a^	0.26 ± 0.02 ^a^
Methylellagic acid ^2^	2.38 ± 0.13 ^a^	0.83 ± 0.03 ^b^	0.70 ± 0.04 ^c^
Total:	17.78 ± 0.77 ^b^	16.86 ± 0.83 ^b^	21.29 ± 1.31 ^a^
Flavonoids (µg/g)			
Kaempferol dihexoside ^3^	Det.	59.88 ± 6.59 ^b^	77.71 ± 7.60 ^a^
Quercetin 3-O-glucoside (Isoquercetin)	28.74 ± 2.00 ^b^	35.67 ± 2.84 ^a^	33.17 ± 2.31 ^a^
Kaempferol 3-O-glucoside (Astragalin)	340.8 ± 7.64 ^c^	968.2 ± 18.32 ^b^	1070.4 ± 10.9 ^a^
Quercetin	Det.	28.99 ± 3.15 ^a^	17.12 ± 1.81 ^b^
Kaempferol	101.5 ± 12.76 ^c^	516.6 ± 52.8 ^a^	370.2 ± 35.20 ^b^
Total:	471.04 ± 21.31 ^b^	1609.34 ± 80.2 ^a^	1568.6 ± 71.39 ^a^
Naphtoquinones (mg/g)			
Plumbagin *	6.71 ± 0.27 ^c^	7.80 ± 0.31 ^b^	8.67 ± 0.26 ^a^
Dihydroplumbagin hexoside ^4^	6.36 ± 0.45 ^c^	9.52 ± 0.52 ^b^	16.3 ± 1.23 ^a^
Total:	13.07 ± 1.02 ^c^	17.32 ± 1.23 ^b^	24.97 ± 2.29 ^a^

^1^—calculation was based on calibration curve for gallic acid; ^2^—calculation was based on calibration curve for ellagic acid; ^3^—calculation was based on calibration curve for kaempferol; ^4^—calculation was based on calibration curve for plumbagin, and the results correspond to the extract obtained immediately after the extraction procedure; *—calculation was carried out in acetone extract; different superscript letters within a row indicate statistically significant differences among means (*p* < 0.05) according to one-way ANOVA followed by Tukey’s post hoc test.

## Data Availability

The dataset is available upon request from the authors.

## Supplementary Materials

The following supporting information can be downloaded at: https://www.mdpi.com/article/10.3390/molecules30183746/s1, Table S1. Calibration data for quantitative analysis. Figure S1. Example of MS and UV-Vis spectrum main components identified in *A. vesiculosa* extracts. Figure S2. Comparison of the UV–Vis spectrum of (a) kaempferol 3-O-glucoside and (b) an unknown flavonoid found in *A. vesiculosa*, as well as the MS spectrum obtained in positive ionization mode. Figure S3. Chromatograms illustrate the completeness of extraction for the isolation of phenolic compounds and plumbagin: (a) Overlaid chromatogram of a two-step extraction using methanol followed by 80% methanol (red line) and the chromatogram after the third-step extraction using 60% methanol (grey line); (b) Overlaid chromatogram of a two-step extraction using acetone (blue line) and the chromatogram after the third-step extraction (grey line). The plumbagin peak was observed at RT = 60.75 min. Figure S4. Overlapped chromatograms of *A. vesiculosa* extract immediately after extraction (red line) and after 24 hours of storage at room temperature (green line), showing the degradation of the labile compound dihydroplumbagin hexoside (RT 5.79 min): a—DAD chromatogram, b—EIC chromatogram with *m/z* = 189 corresponding to the aglycone. The elution conditions were as follows: from 30% acetonitrile to 70% acetonitrile over 60 min. Figure S5. BPC and DAD (λ = 254 nm) chromatograms over a wide time window using gradient as follows: 0–8 min from 98% A to 93% A, 8–15 min from 93% A to 88%, 15–29 min from 88%. A to 85% A, 29–40 min from 85% A to 80% A, 40–50 min from 80% A to 75% A, 50–60 min from 75% A to 55% A, and 60–85 min from 55% A to 100% A (keep constant to 140 min).
