# Supplementary material for: Phenolic Secondary Metabolites in Aldrovanda vesiculosa L. (Droseraceae)
Source: Molecules. 2025 Sep 15;30(18):3746. doi: 10.3390/molecules30183746 (PMC12472234; doi:10.3390/molecules30183746)
Supplement: Supplementary file 1 [file molecules-30-03746-s001.zip › molecules-3853925-supplementary.pdf]

Phenolic Secondary Metabolites in *Aldrovanda vesiculosa* L. (Droseraceae)

Magdalena Wójciak, Ireneusz Sowa, Maciej Strzemski, Marzena Parzymies, Magdalena Pogorzelec, Piotr Stolarczyk and Bartosz Płachno

Table S1. Calibration data for quantitative analysis.

Compound	Calibration range (mg/mL)	equation
Gallic acid	0.002–0.02	y = 32239x – 15.6
Ellagic acid	0.008–0.08	y = 60112x – 103.3
Ellagic acid	0.0005–0.005	y = 43937x – 4.3
Quercetin 3-O-glucoside	0.0001–0.001	y = 27897x – 1.5
Kaempferol 3-O-glucoside	0.00052–0.0052	y = 16987x – 3.1
Quercetin	0.0001–0.001	y = 42961x – 1.3
Kaempferol	0.0003–0.003	y = 26706x – 3.2
Plumbagin	0.003–0.03	y = 40929x + 4.1

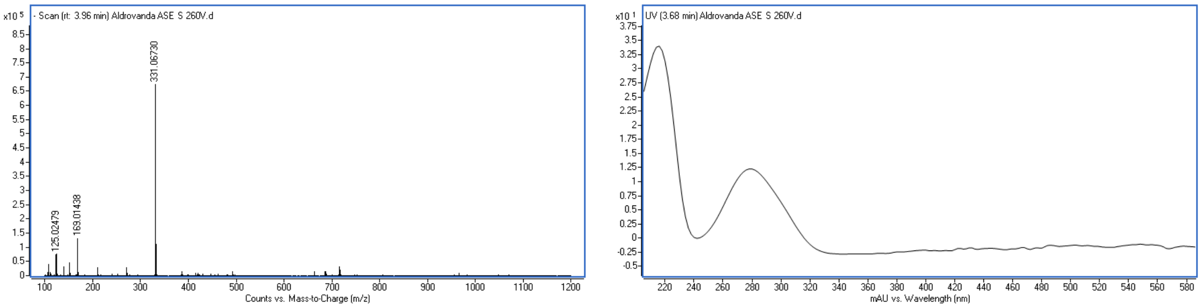

Galloylhexoside

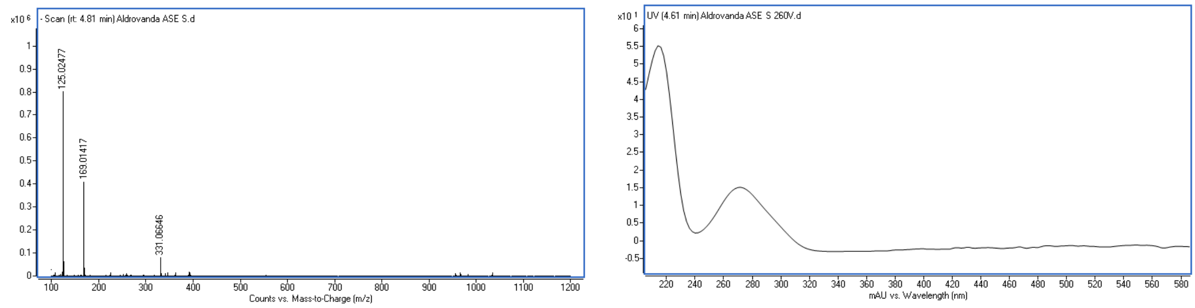

Gallic acid

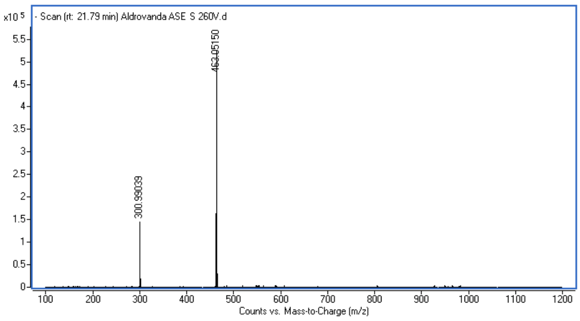

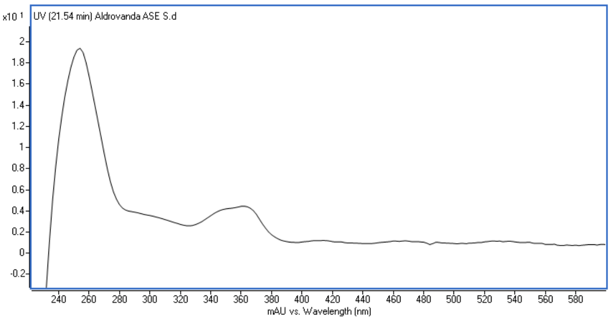

Ellagic acid glucoside

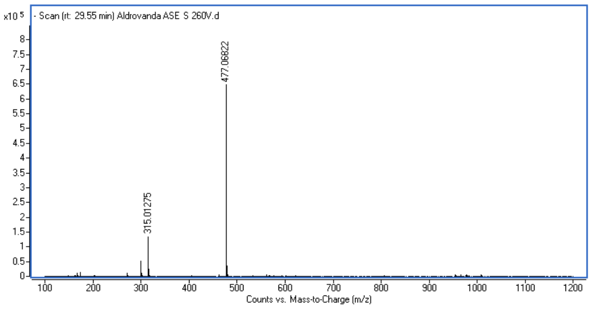

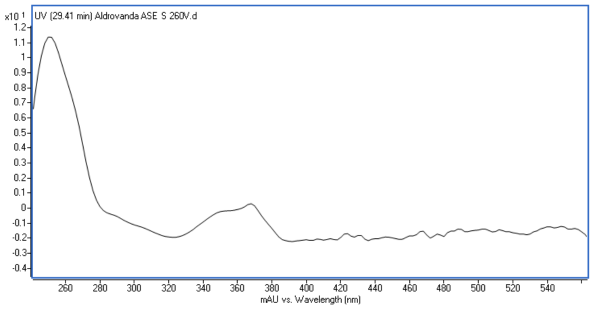

methylellagic acid glucoside

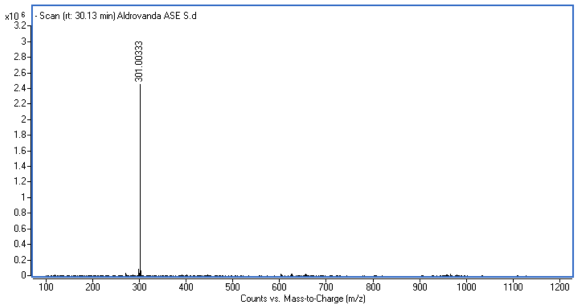

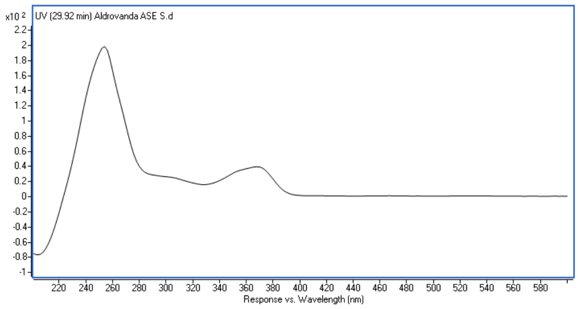

Ellagic acid

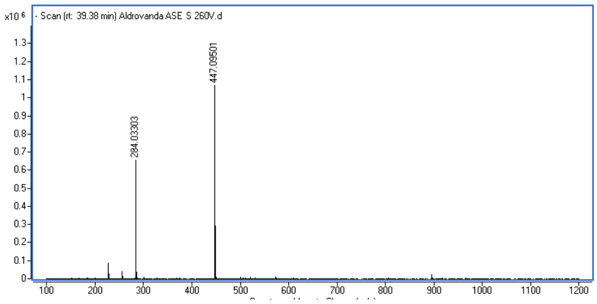

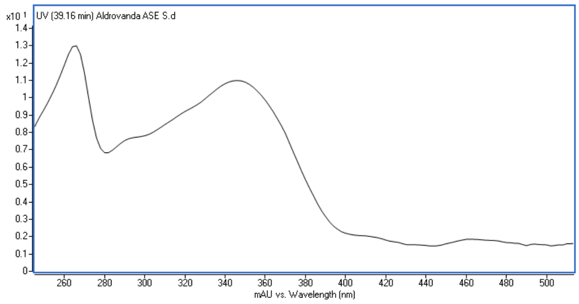

kaempferol 3-O-glucoside

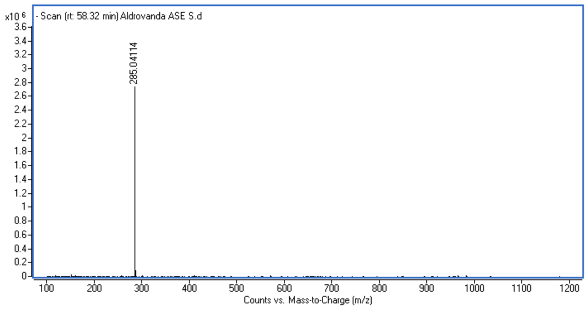

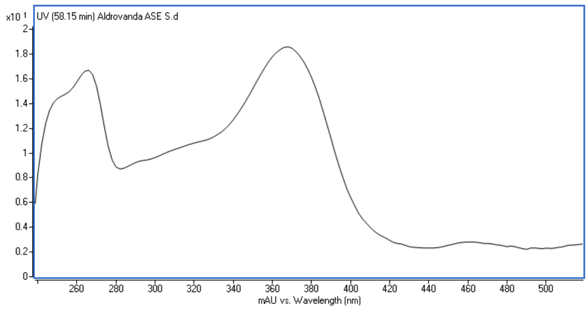

kaempferol

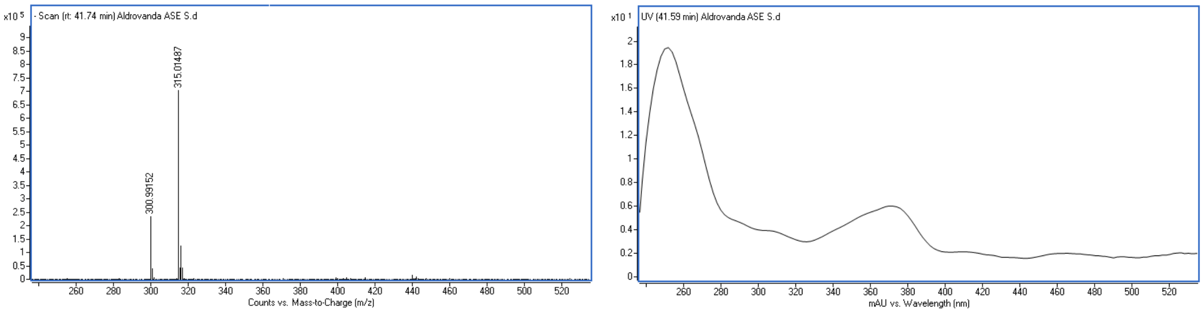

methylellagic acid

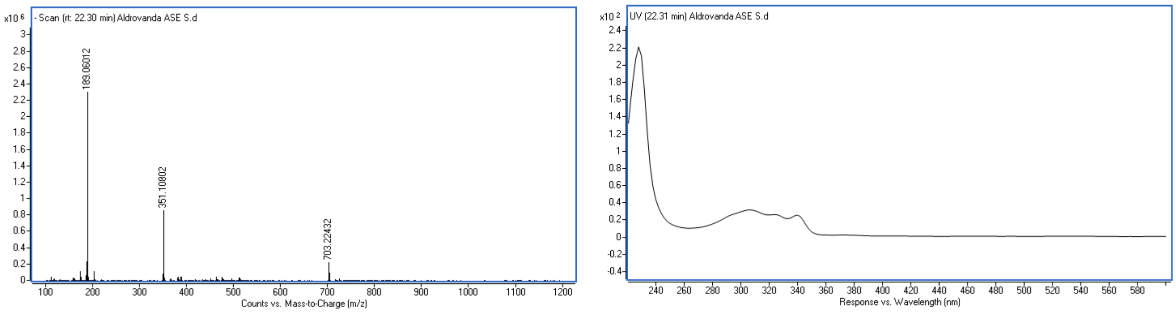

hydroplumbagin glycoside

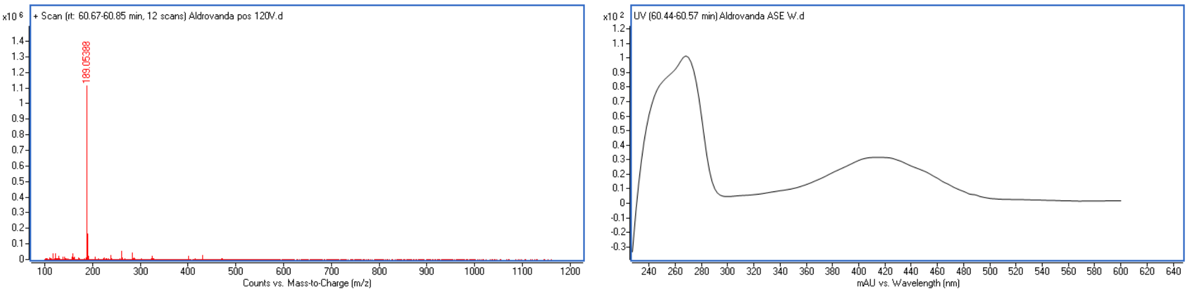

Plumbagin

Figure S1. Example of MS and UV-Vis spectrum main components identified in *A. vesiculosa* extracts.

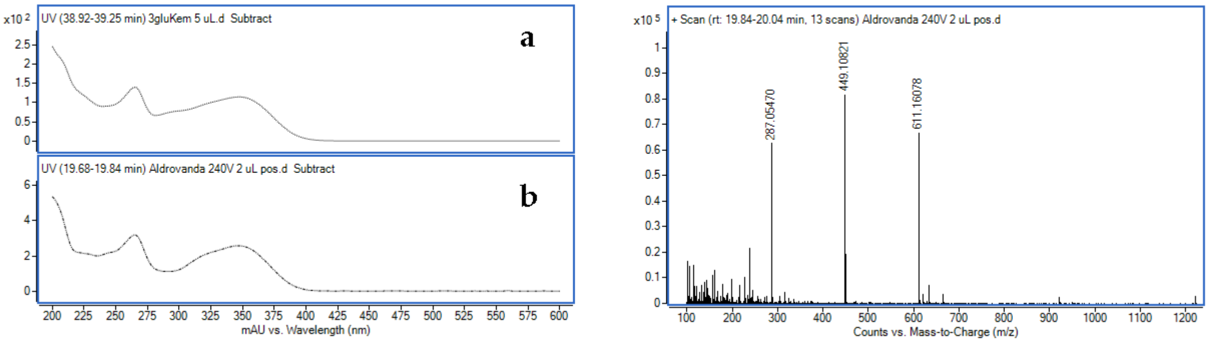

Figure S2. Comparison of the UV–Vis spectrum of (a) kaempferol 3-O-glucoside and (b) an unknown flavonoid found in *A. vesiculosa*, as well as the MS spectrum obtained in positive ionization mode.

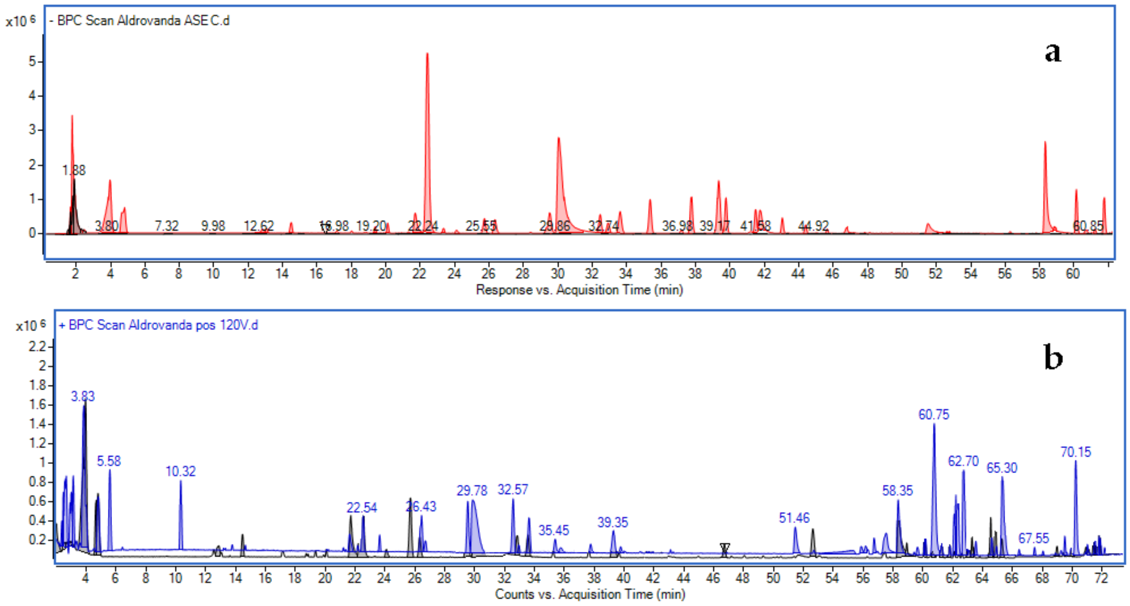

Figure S3. Chromatograms illustrate the completeness of extraction for the isolation of phenolic compounds and plumbagin: a) Overlaid chromatogram of a two-step extraction using methanol followed by 80% methanol (red line) and the chromatogram after the third-step extraction using 60% methanol (grey line); b) Overlaid chromatogram of a two-step extraction using acetone (blue line) and the chromatogram after the third-step extraction (grey line). The plumbagin peak was observed at RT = 60.75 min.

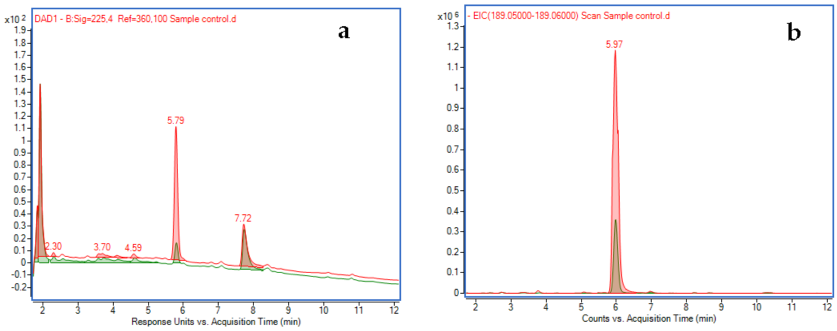

Figure S4. Overlapped chromatograms of *A. vesiculosa* extract immediately after extraction (red line) and after 24 hours of storage at room temperature (green line), showing the degradation of the labile compound dihydroplumbagin hexoside (RT 5.79 min): a – DAD chromatogram, b – EIC chromatogram with m/z = 189 corresponding to the aglycone. The elution conditions were as follows: from 30% acetonitrile to 70% acetonitrile over 60 min.

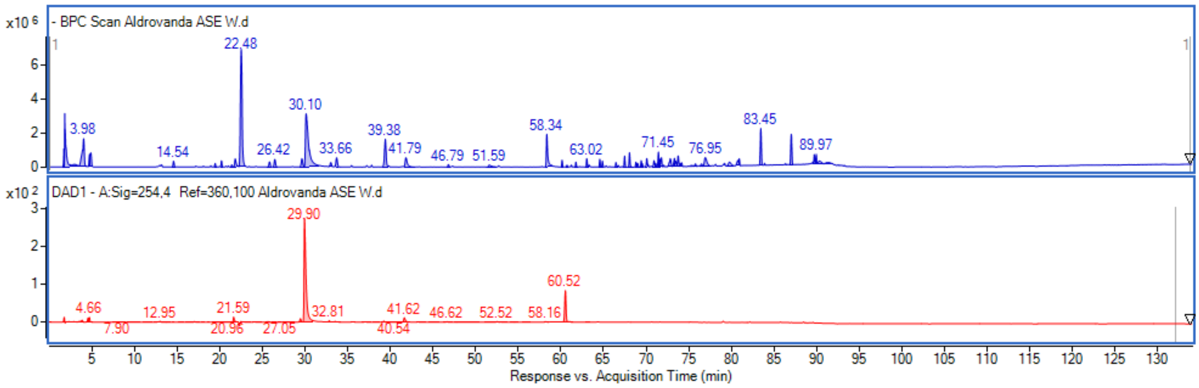

Figure S5. BPC and DAD (λ=254 nm) chromatograms over a wide time window using gradient as follows: 0–8 min from 98% A to 93% A, 8–15 min from 93% A to 88%, 15–29 min from 88%. A to 85% A, 29–40 min from 85% A to 80% A, 40–50 min from 80% A to 75% A, 50–60 min from 75% A to 55% A, and 60–85 min from 55% A to 100% A (keep constant to 140 min.)
